# Celastrol induces apoptosis of gastric cancer cells by miR-146a inhibition of NF-κB activity

**DOI:** 10.1186/1475-2867-13-50

**Published:** 2013-05-27

**Authors:** Min Sha, Jun Ye, Li-xin Zhang, Zheng-yun Luan, Ya-bao Chen

**Affiliations:** 1 Institute of Clinical medicine, Taizhou people’s Hospital affiliated of Nantong University of medicine, 210 Yingchun, Taizhou, Jiangsu Province, 225300, China

**Keywords:** Gastric cancer, Celastrol, miR-146a, NF-κB, Apoptosis

## Abstract

**Background:**

Celastrol, a plant triterpene, is known to play important role in inhibiting proliferation and inducing apoptosis of gastric cancer cells. In the present study, the mechanism of celastrol on gastric cancer cells apoptosis was examined.

**Methods:**

We assessed effect of celastrol on NF-κB signaling pathway in gastric cancer cells using western blot and luciferase reporter assay. The real-time PCR was used to evaluate the effect of celastrol on miR-146a expression, and miR-146a mimic to evaluate whether over-expression of miR-146a can affect NF-κB activity. Finally, the effect of miR-146a on celastrol-induced anti-tumor activity was assessed using miR-146a inhibitor.

**Results:**

Celastrol decreased gastric cancer cells viability in a dose-dependent. Celastrol also reduced IκB phosphorylation, nuclear P65 protein levels and NF-κB activity. Furthermore, Celastrol could increase miR-146a expression and up-regulation of miR-146a expression could suppress NF-κB activity. More important, down-regulation of miR-146a expression can reverse the effect of celastrol on NF-κB activity and apoptosis in gastric cancer cells.

**Conclusions:**

In this study, we demonstrated that the effect of celastrol on apoptosis is due to miR-146a inhibition of NF-κB activity.

## Background

Gastric cancer is one of the most common cancers worldwide, which is particularly high in eastern Asia [1]. Despite the advances in current therapeutic approaches such as surgery, hormone, radio- and chemotherapy, gastric cancer is still the second most common cause of death from cancer [2]. Therefore, it is urgent to make clear the key mechanism of the apoptosis in gastric cancer as well as establishment of the appropriate blocking channels is important for the development of more effective therapies of gastric cancer.

Celastrol is a plant triterpene derived from the root of Thunder of God Vine and can resist various inflammatory and neurodegenerative disorders [3-5]. It has been reported that celastrol plays important role in inhibiting proliferation and inducing apoptosis of wide variety of human tumor cell types including multiple myeloma, hepatocellular carcinoma, gastric cancer and so on [6]. However, its mechanism is not well understood.

MicroRNAs (miRNAs) are a small, non-coding RNA involved in post-transcriptional gene regulation by binding to a target site in the 3′-UTR of target mRNAs [7]. Recent evidence has revealed that the change of miRNAs expression was associated with tumorigenesis, which indicated that miRNAs could act as tumor suppressors or oncogenes [8,9]. One of these microRNAs, miR-146a is down-regulated in gastric cancer, and induces apoptotic effects by inhibiting G protein-coupled receptor-mediated activation of NF-κB [10,11]. When the expression of miR-146a was up-regulated in human gastric cancer, it was showed to induce cell apoptosis.

The transcription factor NF-κB is activated in tumorgenic processes including gastric cancer and may be an important pharmacological target for this disease [12,13]. Activation of NF-κB predominantly occurs via the release of the p50/p65 heterodimer from the inhibitor of κB (IκB) complex in the cytosol of the cells. When released from IκB, the p50/p65 heterodimer translocates to the cell nucleus and regulates downstream gene expression [14]. In recent years, more and more evidence indicates that celastrol can induce apoptosis of various tumors by inhibiting of NF-κB activation [15,16]. As a result, the aim of this study was to evaluate whether miR-146a regulating NF-κB activity is involved in apoptosis of human gastric cancer cells induced by celastrol.

## Results

### Celastrol repressed cell viability of gastric cancer cells and NF-κB signaling pathway

**Figure 1:**
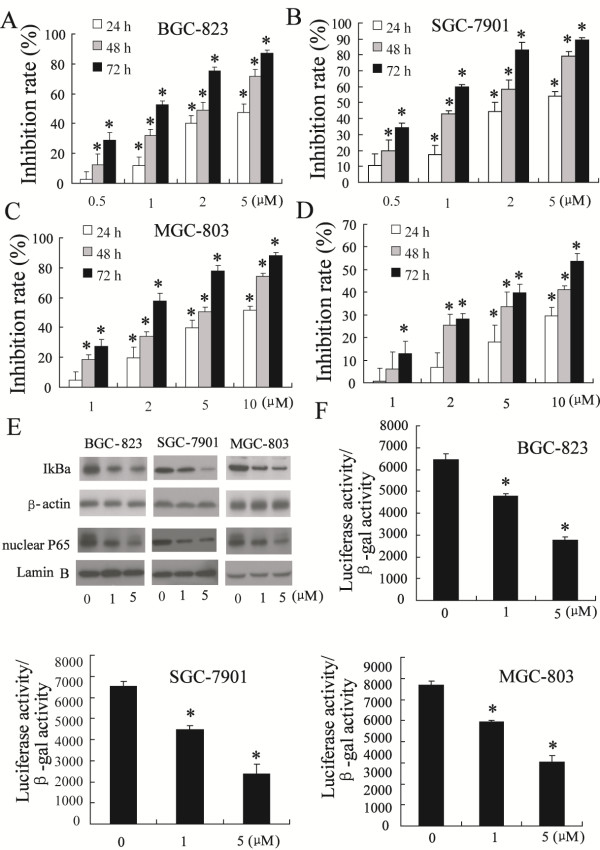
**Celastrol repressed viability of gastric cancer cells and NF-κB signaling pathway.** Exposure to various concentrations of celastrol resulted in dose- and time-dependent growth inhibition of BGC-823 cells (**A**), SGC-7901 cells (**B**), MGC-803 cells (**C**) and GES-1 cells (**D**). Celastrol inhibited phosphorylation of IκB and nuclear p65 content in dose-dependent manner (**E**). Celastrol decreased NF-κB transcriptional activity in BGC-823 cells, SGC-7901 cells and MGC-803 cells in a dose-dependent manner (**F**). *P < 0.05, indicate significant differences from the respective control groups.

The inhibitory effects of celastrol on the growth of gastric cancer cells and normal gastric epithelial cells were evaluated using MTT assays. Celastrol treatment inhibited the growth of BGC-823, SGC-7901 and MGC-803 cells in a dose- and time-dependent manner (Figure 1A, 1B and 1C). And the IC 50 value at 72 h after treatment was 0.989 μM (BGC-823 cells), 0.798 μM (SGC-7901 cells) and 1.98 μM (MGC-803 cells). Normal gastric epithelial cells showed more resistance to the cytotoxicity effect of celastrol. And the IC 50 value at 72 h was 8.16 μM for GES-1 cells (Figure 1D).

Next, we wanted to determine whether the NF-κB pathway was inhibited by celastrol in gastric cancer cells. Therefore, phosphorylation of IκB was investigated as a potential response to treatment with celastrol. As shown by western blot analysis in Figure 1E, celastrol inhibited phosphorylation of IκB in a dose-dependent manner in BGC-823 cells, SGC-7901 cells and MGC-803 cells.

It is well known that the phosphorylation and degradation of IκB can result in the release of NF-κB complex followed by its rapid translocation into the nucleus [17]. To investigate the nuclear entry of NF-κB complex, the p65 content was measured by western blot in BGC-823, SGC-7901 and MGC-803 cells. Treatment with celastrol could inhibit p65 entry into the nucleus (Figure 1E). The inhibition of NF-κB signaling pathway by celastrol was also determined by the observation of the transcriptional activity of NF-κB. As shown in Figure 1F, the treatment of BGC-823, SGC-7901 and MGC-803 cell with celastrol strongly inhibited the NF-κB transcriptional activity in a dose-dependent manner.

### Celastrol increases miR-146a expression

**Figure 2:**
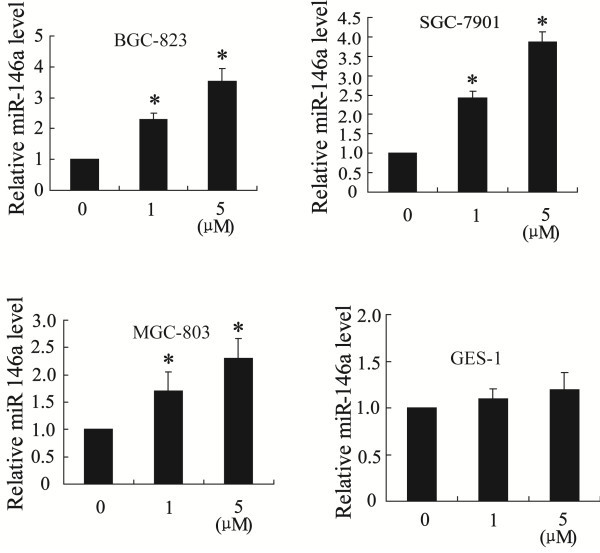
**Celastrol stimulates miR-146a expression in gastric cancer cells.** The expression of miR-146a was significantly increased after celastrol treatment for 12 h in BGC-823, SGC-7901 and MGC-803 cells. *P < 0.05 vs. control group.

To further identify the mechanism for celastrol inhibition of BGC-823, SGC-7901 and MGC-803 cells growth, we performed real-time PCR to detect miR-146a expression after treated with 2 μM of celastrol for 12 h. As shown in Figure 2, celastrol treatment significantly increased the expression of miR-146a in these cells (*P < 0.01*). However, celastrol can not significantly induce miR-146a expression in GES-1 cells.

### Up-regulation of miR-146a expression can inhibit NF-κB activity

**Figure 3:**
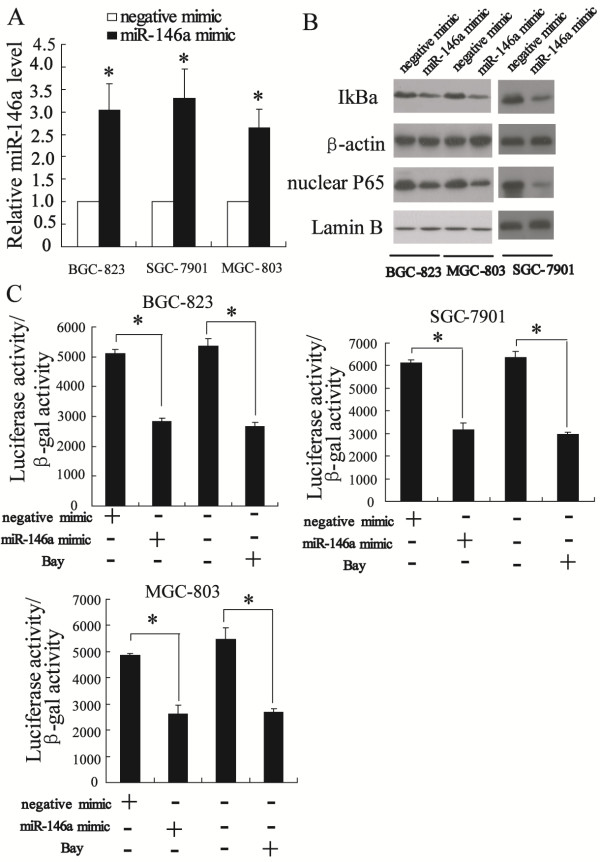
**Up-regulation of miR-146a expression can inhibit NF-κB activity in BGC-823, SGC-7901 and MGC-803 cells.** The real-time PCR revealed that miR-146a mimic significantly increased the expression of miR-146a (**A**). Western blot showed that transfected miR-146a mimic decreased phosphorylation of IκB and nuclear p65 level (**B**). The luciferase reporter assay revealed that transfected miR-146a mimc significantly repressed NF-κB transcriptional activity (**C**). *P < 0.05, indicate significant differences from the respective control groups.

To determine whether miR-146a can regulate signaling pathway in gastric cancer cells, we investigated the modulation of phosphorylation of IκB in cells transfected miR-146a mimic by western blot. The result of real-time PCR revealed that miR-146a mimic can significantly increase the expression of miR-146a in BGC-823, SGC-7901 and MGC-803 cells (*P < 0.01*) (Figure 3A), suggesting that miR-146a mimic is efficiently introduced into the cells and acts to up-regulate miR-146a expression. Furthermore, transfection of miR-146a mimic decreased phosphorylation of IκB and nuclear P65 as shown in Figure 3B.

We also measured the transcriptional activity of NF-κB using Luciferase reporter assay. Figure 3C showed that up-regulation of miR-146a expression decreased NF-κB activity, which was similar to Bay.

### Down-expression of miR-146a can reverse the effect of celastrol on NF-κB activity and apoptosis

**Figure 4:**
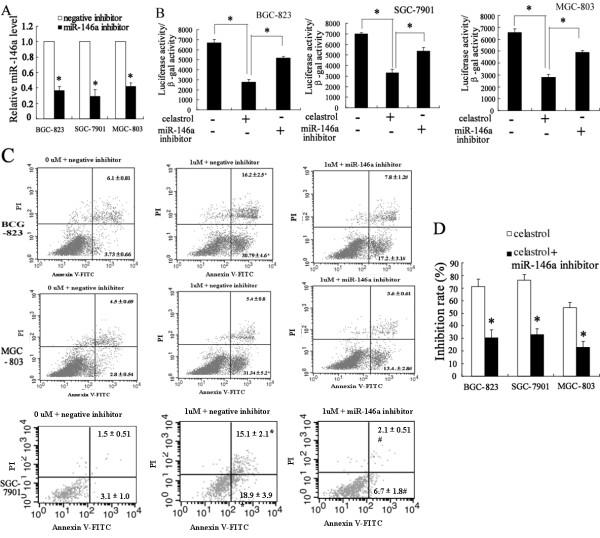
**Down-expression of miR-146a can reverse the effect of celastrol on NF-κB activity and apoptosis.** The real-time PCR revealed that miR-146a inhibitor can significantly decrease the expression of miR-146a in BGC-823, SGC-7901 and MGC-803 cells (**A**). After transfected with 100 nmol/L of miR-146a inhibitor, the cells were treated with 2 μM celastrol for 6 h. miR-146a inhibitor significantly increased NF-κB transcriptional activity in BGC-823, SGC-7901 and MGC-803 cells after celastrol treatment (**B**). Flow-cytometric analysis showed that miR-146a inhibitor significantly decreased the apoptosis of BGC-823, SGC-7901 and MGC-803 cells induced with celastrol (**C**). After transfected with 100 nmol/L of miR-146a inhibitor, the cells were treated with 2 μM celastrol for 72 h. MTT revealed that miR-146a inhibitor significantly decreased the effect of celastrol on inhibition of BGC-823, SGC-7901 and MGC-803 cells growth (**D**). *P < 0.05, indicate significant differences from the respective control groups.

In order to evaluate the role of miR-146a in the effect of celastrol on inhibition of BGC-823, SGC-7901 and MGC-803 cells apoptosis, we treated cells with celastrol after transfected with miR-146a inhibitor. As shown in Figure 4A, miR-146a inhibitor can significantly decrease the expression of miR-146a in BGC-823, SGC-7901 and MGC-803 cells (*P < 0.01*). Interestingly, we found that celastrol treatment inhibited transcriptional activity of NF-κB, which can be restored by inhibition of miR-146a expression (Figure 4B). In addition, the number of cells that underwent apoptosis significantly increased after treated with 2 μM celastrol. Moreover, the celastrol induced apoptosis was attenuated by miR-146a inhibitor in BGC-823, SGC-7901 and MGC-803 cells (Figure 4C). Furthermore, BGC-823, SGC-7901 and MGC-803 cells growth was increased in celastrol-treated cells after transfected miR-146a inhibitor (Figure 4D).

## Discussion

The present report describes the molecular mechanism of growth arrest induced by celastrol in human gastric cancer cells. Celastrol inhibited proliferation of BGC-823, SGC-7901 and MGC-803 cells, but had little effect on GES-1 cells, indicating celastrol induces selective cytotoxicity in cells. This triterpene caused the down-regulation of phosphorylation of IκB and nuclear P65 protein levels, and it inhibited transcriptional activity of NF-κB. Celastrol increased miR-146a expression in gastric cancer cells and overexpression of miR-146a can significantly decrease NF-κB activity. Further, it induced apoptosis of BGC-823, SGC-7901 and MGC-803 cells, which can be reversed by down-regulation of miR-146a expression. We also found that celastrol inhibited NF-κB activity could also be restored by down-regulation of miR-146a expression.

Increasing evidence showed that celastrol can inhibit tumor cell proliferation and induce apoptosis through inhibition of NF-kB activity [18,19]. Activation of the classical pathway of NF-κB involves the phosphorylation and subsequent degradation of IκBα, leading to the release and translocation of NF-κB to the nucleus [20]. Our studies showed that in gastric cancer cells, celastrol was observed to decrease phosphorylation of IκB and subsequent inhibition of NF-κB signaling pathway, exhibiting an anti-tumor potential. More important, the IC 50 value at 72 h after treatment was 0.989 μM for BGC-823 cells 0.798 μM for SGC-7901 cells and 1.98 μM for MGC-803 cells, but 8.16 μM for GES-1 cells. These results indicated that celastrol could play the role in killing gastric cancer cells without destruction to the normal cells, which is in agreement with the previous reports [6].

To further clarify the mechanism involved in the inhibition of viability of gastric cancer cells, we investigated the effect of celastrol on the expression of miR-146a. Prior study indicated that miR-146a is down-regulated in gastric cancer tissue samples and cell lines and overexpression of miR-146a could induce apoptosis of cancer cells [10,11]. As a result, miR-146a may be a promising target for treatment of gastric cancer. Interestingly, celastrol was observed to up regulate the expression of miR-146a in BGC-823, SGC-7901 and MGC-803 cells but not in GES-1 cells, which indicated that miR-146a may be involved in growth inhibition induced by celastrol. This hypothesis was confirmed that inhibition of miR-146a expression can reverse the effect of celastrol on apoptosis and growth inhibition of cancer cells.

We also found that over-expression of miR-146a could reduce phosphorylation of IκB and nuclear P65 protein levels, leading to inhibition of NF-κB transcriptional activity. Moreover, inhibition of miR-146a expression can reverse NF-κB activity repression by celastrol. According to these results, we draw a conclusion that celastrol suppresses NF-κB activity by enhancing miR-146a expression.

In summary, we established a primary relationship between celastrol and apoptosis in BGC-823, SGC-7901 and MGC-803 cells here. Furthermore, we explored the molecular mechanisms of celastrol-induced apoptosis in gastric cancer cells. These results suggest that celastrol might be an effective drug to treat human gastric cancer.

## Materials and methods

### Chemicals and reagents

Dulbecco’s modified Eagle’s medium (DMEM), sodium pyruvate were from Gibco-BRL (Rockville, MD, USA). Fetal bovine serum (FBS) was from PAA Laboratories (GmbH, Linz, Austria). Celastrol was purchased from Alexis Biochemicals (San Diego, CA). Bay 11–7082 (Bay) and dimethylthiazoly-2, 5-diphenyltetrazolium bromide (MTT) were obtained from Sigma Aldrich. Mouse anti-phospho-IκB (Ser32/36) antibody was obtained from Cell Signaling Technology (Beverly, MA, USA). Mouse anti-β-actin and goat anti-Lamin B antibodies were purchased from Santa Cruz Biotechnologies (Santa Cruz, CA, USA). Annexin V-EGFP ⁄ PI Apoptosis Detection Kit was from KeyGen (Nanjing, China). The Detergent Compatible (DC) Protein Assay kit was purchased from Bio-Rad Laboratories (Hercules, CA, USA). The miRNeasy Mini kit, the miScript Reverse Transcription kit and the miScript SYBR Green PCR kit were purchased from Qiagen (Hilden, Germany).

### Cell culture

Human gastric adenocarcinoma cell line BGC-823 cells and SGC-7901 cells, and gastric mucinous adenocarcinoma cell line MGC-803 were purchased from the Shanghai Institute of Cytobiology in Chinese Academy of Sciences. Human normal gastric epithelial cell line GES-1 was obtained from the Institute of Oncology in Beijing University. The cells were grown in DMEM medium supplemented with 10% FBS, 100 units/ml penicillin and 100 μg/mL streptomycin, and routinely passaged at 3-day intervals. All cell lines were maintained at 37°C in a humidified incubator containing 5% CO_2_ and treated with celastrol (dissolved in DMSO) in complete DMEM medium. To obtain reliable results, the final concentration of DMSO in the culture medium was kept less than 0.1%.

### MTT assay

Cell viability was determined using MTT [3-(4,5-dimethylthiazol-2-yl)-2,5-diphenyltetrazolium bromide assays. Briefly, the cells were seeded in 96-well dishes at 1 × 10^4^ cells per well, and treated with different concentrations of celastrol. Then each well was supplemented with 10 μL MTT and incubated for 4 h at 37°C. The medium was then removed, and 150 μL DMSO (Sigma Aldrich) was added to solubilize the MTT formazan. The optical density was read at 490 nm. The inhibition rate was calculated as follows: inhibition rate = [(mean control absorbance - mean experimental absorbance) ⁄ mean control absorbance] × 100 (%). To evaluate the effect of celastrol on cellular growth, the concentration that caused 50% growth inhibition (IC 50) was calculated by the modified Karbers method according to the formula: IC50 = log^− 1^[X_k_ − i (∑P  − 0.5)], in which X_k_ represents the celastrol of the highest drug concentration; i is that of ratio of adjacent concentration; and ∑P is the sum of the percentage of growth inhibition at various concentrations [21].

### Flow cytometry analysis

This assay is based on the translocation of phosphatidylserine from the inner leaflet of the plasma membrane to the cell surface in early apoptotic cells [22]. Briefly, cells were resuspended in a binding buffer. Next, annexin V-EGFP and PI were added and the solution was incubated at room temperature for 15 min in the dark, followed by assay on FACScan (Becton Dickin-son). The percentage of apoptosis was computed using Cell-Quest software (Becton Dickinson).

### Oligonucleotides and Cell Transfection

miR-146a was knocked down or overexpressed by transfection with miRNA inhibitor or miRNA mimic using siPort Neo-FX (Ambion) according to the manufacturer’s recommendations. miR-146a mimics (5′-UGAGAACUGAAUUCCAUGGGUU-3′), miR-146a inhibitor (5′-AACCCAUGGAAUUCAGUUCUCA-3′) and negative mimics/inhibitors (NC, 5′-UUGUACUACACAAAAGUACUG-3′) were synthesized by RIBOBIO (Ribobio Co. Ltd, Guangzhou, China). All of the oligonucleotides were transfected at a final concentration of 100 nM. BGC-823, SGC-7901 and MGC-803 cells were transfected with miR-146a inhibitor or mimic using siPort Neo-FX (Ambion) according to the manufacturer’s recommendations.

### Quantitative real-time PCR (Q-PCR) analysis of miRNA expression

Approximately 5 × 10^6^ cells were treated without (control) or with celastrol for 12 h. miRNAs were isolated and purified using Trizol reagent (Invitrogen, USA), according to manufacturer’s protocol. The miR-146a level was quantified by real-time PCR using TransStartTM SYBR Green qPCR Supermix (TransGen Biotech, Beijing, China), and with U6 small nuclear RNA as an internal normalized reference. For miR-146a, the primers were as follows: forward, 5′- GCCGATTGGAGTGGTAAAC-3′ and reverse, 5′-AAGACCCCTCGTTGCAGT-3′. For U6, the primers were as follows: forward, 5′-CTCGCTTCGGCAGCACA-3′ and reverse, 5′-AACGCTTCACGAATTTGCGT-3′.

### Luciferase reporter assay

The reporter plasmid, pNF-κB-luc, containing the κB-enhancer consensus sequences [(TGGGGACTTTCCGC) × 5] and NF-κB-dependent firefly luciferase gene was purchased from Stratagene (La Jolla, CA, USA). BGC-823, SGC-7901 and MGC-803 cells were transiently transfected with two plasmids (pNF-κB-luc plasmid and β-galactosidase) using the LipofectAMINE Plus regent. Cells were passaged on 24-wells plate the day prior to transfection to achieve 80%-85% confluence on the following day. Twelve hours after transfection, cells were incubated for an additional 24 h in medium containing different concentration of celastrol and harvested for luciferase reporter assays. Luciferase activity was measured with a luminometer (TD-20/20; Turner Designs, CA, USA) using the Luciferase Assay System. β-Galactosidase activity was detected to normalise any variations in the transfection efficiency.

### Western blot analysis

Total protein was extracted using a lysis buffer containing 50 mmol/l Tris–HCl, pH 7.4; 1% NP-40; 150 mmol/l NaCl; 1 mmol/l EDTA; 1 mmol/l phenylmethylsulfonyl fluoride; and complete proteinase inhibitor mixture (one tablet per 10 ml; Roche Molecular Biochemicals, Indianapolis, IN, USA). The cytosol and nuclear extracts were prepared by NE-PER nuclear and cytoplasmic extraction reagents (Pierce Biotechnology, Rockford, IL, USA) according to the manufacturer’s instructions. Protein concentration in the cell lysate was quantified using the DC protein assay kit (Bio-Rad). Then, Western blot analysis was performed. β-actin and Lamin B were used as internal controls for the total and nuclear extracts, respectively.

### Statistical analysis

Statistical analysis was performed with statistical analysis software SPSS 13.0 software. Statistical analyses were performed using either an analysis of variance (ANOVA) or Student’s *t*-test. Data were expressed as mean ± standard deviation. *P* < 0.05 was considered to be significant.

## Competing interests

The authors report no conflicts of interest.

## Authors’ contributions

MS carried out the molecular genetic studies and provided conception and design. JY participated in data acquisition. LZ participated in the sequence alignment. ZL contributed to statistical analysis. YC drafted the manuscript and participated in data analysis and interpretation. All authors read and approved the final manuscript.
